# Entropy and Multifractal-Multiscale Indices of Heart Rate Time Series to Evaluate Intricate Cognitive-Autonomic Interactions

**DOI:** 10.3390/e23060663

**Published:** 2021-05-25

**Authors:** Pierre Bouny, Laurent M. Arsac, Emma Touré Cuq, Veronique Deschodt-Arsac

**Affiliations:** 1 Univ. Bordeaux, CNRS, Laboratoire IMS, UMR 5218 Talence, France; laurent.arsac@u-bordeaux.fr (L.M.A.); veronique.arsac@u-bordeaux.fr (V.D.-A.); 2 URGOTECH, 15 avenue d’Iéna, 75116 Paris, France; etoure@urgotech.fr

**Keywords:** HRV, complexity, entropy, multifractality, cognitive state

## Abstract

Recent research has clarified the existence of a networked system involving a cortical and subcortical circuitry regulating both cognition and cardiac autonomic control, which is dynamically organized as a function of cognitive demand. The main interactions span multiple temporal and spatial scales and are extensively governed by nonlinear processes. Hence, entropy and (multi)fractality in heart period time series are suitable to capture emergent behavior of the cognitive-autonomic network coordination. This study investigated how entropy and multifractal-multiscale analyses could depict specific cognitive-autonomic architectures reflected in the heart rate dynamics when students performed selective inhibition tasks. The participants (N=37) completed cognitive interference (Stroop color and word task), action cancellation (stop-signal) and action restraint (go/no-go) tasks, compared to watching a neutral movie as baseline. Entropy and fractal markers (respectively, the refined composite multiscale entropy and multifractal-multiscale detrended fluctuation analysis) outperformed other time-domain and frequency-domain markers of the heart rate variability in distinguishing cognitive tasks. Crucially, the entropy increased selectively during cognitive interference and the multifractality increased during action cancellation. An interpretative hypothesis is that cognitive interference elicited a greater richness in interactive processes that form the central autonomic network while action cancellation, which is achieved via biasing a sensorimotor network, could lead to a scale-specific heightening of multifractal behavior.

## 1. Introduction

Recent advances conceiving physiological systems as dynamically coordinated networks have deepened our understanding of critical functions that emerge from interdependencies between brain, bodily organs and environment. The main intuition is that large and flexible networked systems arise, re-organize themselves as a function of sensory inputs, mood and internal processing to cohere in an efficient executive control. This way, rather than conceiving independent encapsulated entities, system theories plead for elaborated architectures of intricate networks where interdependencies, governed by nonlinear processes, are more important than the components themselves, to achieve efficient functions. Obviously enough, the studied interactions span several spatial (from neurons to systems) and temporal (from milliseconds to minutes) scales to support adequate regulations, which lead researchers to envisage multiscale nonlinear approaches to obtain reliable metrics for exploring the system complexity. The next intuitive step is that in response to any challenge for the mind or the body, an adequate response necessarily involves a system re-organization, which could be reflected in the output signal dynamics, and captured by said metrics. Hence, advances in the development of nonlinear tools for exploring behavior time series have been an active part of research in complex system investigations [1,2,3,4] and have demonstrated that complex dynamics could be identified beyond the confines of the brain.

Among concrete descriptions of such complex systems, it has been argued for years that a functional network involving a cortical and subcortical circuitry regulating both cognition and cardiac autonomic interactions can be identified. Since Benarroch paved the way in the 1990’s [5] by describing a “central autonomic network” (CAN) that evokes the role of cortical and subcortical connections in integrated visceral control, Thayer and Lane extended the architecture of the CAN by including modulations of brainstem mechanisms associated to vagal-mediated cardiac autonomic control [6]. Possible extensions have been described recently [7], which further demonstrated the richness of this intricate network and multiple interdependencies that take place around this neurovisceral integration around bottom-up and top-down vagal-mediated information between the brain and the heart [8]. Cognitive and emotional modulations are supposed to aggregate coordinated interdependencies, which is the object of recent research based on nonlinear analyses of the temporal structure of heartbeat time series.

The richness of information flow in heart rate time series has been reliably accounted for by entropy metrics in recent research [9,10]. Multiscale entropy (MSE) (and its variants) is a widely used metric to explore signal complexity that has demonstrated substantial interest when applied to heart rate time series [1,3]. The MSE algorithm may encounter problems when applied to short time series analysis [11]. Unfortunately, short series are common in physiological experiments, especially, when participants are subjected to cognitive tasks that can induce fatigue and disengagement if continued for too long. Improvements in validity and accuracy are significant when using, e.g., the refined composite multiscale entropy (RCMSE) or linear multiscale entropy (LMSE, [12]), which offers a reliable tool to explore cognitive-autonomic interactions in comfortable experimental conditions. Recent investigations have suggested that RCMSE applied to heart rate time series is sensitive enough to capture richer cognitive-autonomic interactions during a cognitive task [13] as well as their degradation by stress induction [14]. In addition, RCMSE appeared pivotal in interpreting the benefits of biofeedback training, a practice where the intentional reduction of the breathing rate allowed reaching resonance between sympathetic and vagal oscillators, which enhances the vagal bottom-up stimulation of the frontal cortex and has therapeutic virtues [15].

Subtle changes in the cardiovascular complexity have also been addressed based on multifractal formalisms. Scale-free, fractal behavior has mainly been observed in large scales of heart rate time series; but the recent combination of a multifractal and a multiscale approach offers a new window to reveal the unexpected richness of details in the self-similarity structure in heart rate dynamics [16,17]. This approach nicely exploits the formalism of detrended fluctuation analysis to include multifractal and multiscale computational roots and has been called multifractal-multiscale detrended fluctuation analysis (MM-DFA). The degree of multifractality in the sensorimotor variability has recently been associated with effective system adaptation [18]. As these experiments in the neuromotor domain bring about a brand-new support of the link between the complexity in brain neural dynamics and multifractal output variability, little is known to date on the capacity of multifractal multiscale metrics to capture subtle adaptations in cognitive-autonomic interactions.

The present study aimed at exploring the reliability of RCMSE and MM-DFA metrics, together with additional and surrogate testing, to decipher adaptations in cognitive-autonomic interdependencies when humans are exposed to cognitive tasks. Three cognitive tasks were purposely selected to impose cognitive interference, action cancellation and action restraint. We hypothesized that cognitive interference might be associated to heighten the entropy in heart rate time series as illustrated in previous cognitive testing [13]. On the other hand, simpler reaction tasks are expected to have much less consequences on entropy. Yet, action restraint differs from action cancellation by the need to bias the prepotent sensorimotor system which might be reflected in a higher degree of multifractality.

## 2. Materials and Methods

### 2.1. Population

Thirty-seven trained students of Bordeaux university (19 males,18 females, aged 22.1 ± 1.7 years old) gave their informed consent to take part in the present study that was part of their academic curriculum and for which they received credits. All the procedures were approved by the IRB of the faculté des STAPS and follow the rules of the Declaration of Helsinki. Because of corrupted data in heart rate recordings, three participants were discarded.

All participants reported having no history of neurological or physiological disorders, and normal or corrected-to-normal vision. Participants were asked to avoid alcohol, caffeine and to abstain from heavy physical activity for the 12 h preceding the experiment.

### 2.2. Experimental Design

During the whole experiment lasting about 40 min, the participants remained quietly seated on a chair, at a viewing distance of about 0.6 m of the 15-inch screen of a laptop, whose keyboard was used to respond to the stimuli displayed on the screen. The participants wear a bipolar electrode transmitter belt Polar H10 (Polar, Finland) fitted around their chest and connected to a smartphone via Bluetooth. The cardiac interbeat time intervals (RR) were recorded when needed (see details below).

The experimental setup consisted in four successive phases. The participants first conducted a resting state phase named baseline, during which they watched a neutral animal documentary for 10 min, wearing headphones. Then, three cognitive tasks were assigned to be performed successively in a pseudo-randomized order (approximately 8 min per task and 1 min pause between the tasks), namely Stroop Color and Word Test (SCWT), go/no-go task (GNGT) and stop signal task (SST).

### 2.3. Cognitive Tasks

Executive function tasks were implemented using the software PsyToolkit [19,20].

All stimuli were presented in the center of the screen displaying a black background color. Whatever the task, each trial began with the presentation of a fixation cross that was replaced by the task-specific stimulus (see below for each task) after 250 ms.

Prior to each task, clear explanations were provided; a trial was performed so that the participant becomes aware of the instructions and could find a comfortable way to hit the specified keyboard keys as quickly as possible (lying fingers on the keys).

#### 2.3.1. Stroop Color and Word Task (SCWT)

**Figure 1:**
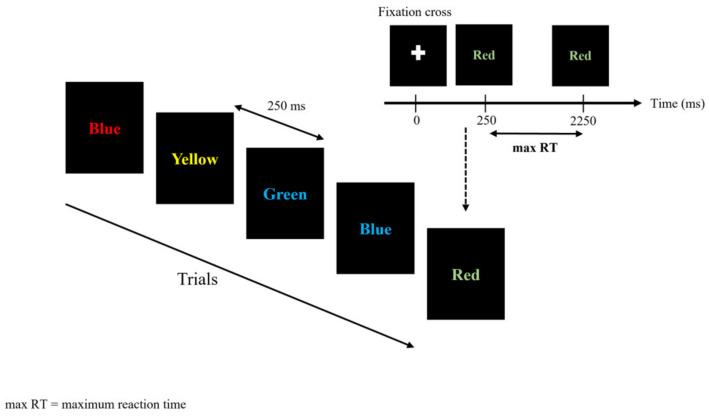
Stroop Color and Word Task (SCWT). Displayed sequences for the SCWT.

This task assesses the ability to inhibit cognitive interference, which occurs when the processing of a stimulus feature affects the simultaneous processing of another attribute of the same stimulus [21]. During the Stroop task, a word naming a color among “blue”, “green”, “red”, or “yellow” appeared in an unpredictable manner at the center of the screen, displayed either in a congruent (similar) color or in incongruent (different) color (blue, green, red, or yellow). To get non-verbal answers from the participants, four keys on the keyboard were tagged with colored stickers. The participants had to hit the keyboard key corresponding to the “ink” color, not the word, as accurately as possible. A stimulus remained displayed on the screen for a maximal duration of 2000 ms (Figure 1). The next stimulus appeared 250 ms after either the response of the participant or an elapsed display time. This task consisted in 400 successive trials.

#### 2.3.2. Go/No-Go Task (GNGT)

**Figure 2:**
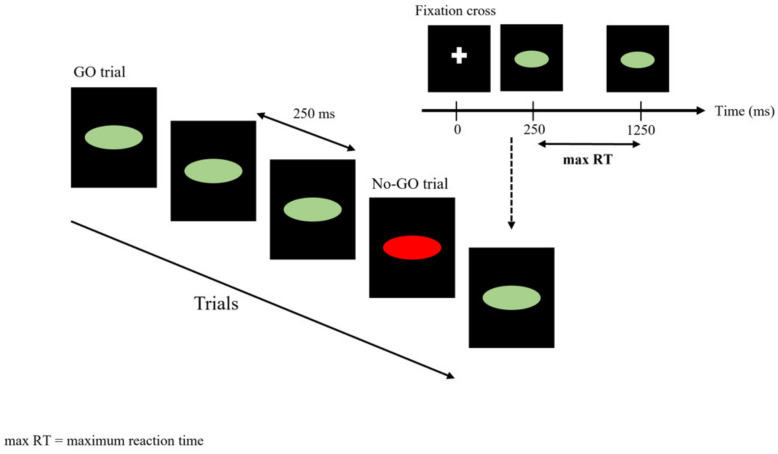
Go/no-go task (GNGT). Displayed sequences for the GNGT. In this task, the participants respond as rapidly as possible to a go stimulus. In a minority of trials (30%), the go stimulus is replaced by a no-go stimulus that instructs participants to not respond.

The go/no-go task (GNGT) measures the participants’ capacity for sustained attention and response control. GNGT captures action restraint thanks to the relative amounts of no-go signals (here 30%) appearing instead of go. Subjects are presented with a series of stimuli and are told to respond when a go stimulus is presented and to withhold their response when a no-go stimulus is presented. The task was set up as follows (Figure 2): an ellipsis appeared at the center of the screen as a stimulus, either with a green background with instruction “press the space bar” (go) or a red background with the instruction “do not press the space bar” (no-go). The participants were instructed to respond (hit keyboard space bar) as quickly as possible to the go signal that in any case remained displayed on the screen for a maximal duration of 1000 ms. After either the response of the participant or an elapsed display time, the next stimulus appeared 250 ms later. The task consisted of 600 successive stimuli including 70% go and 30% no-go, presented in an unpredictable manner.

#### 2.3.3. Stop Signal Task (SST)

**Figure 3:**
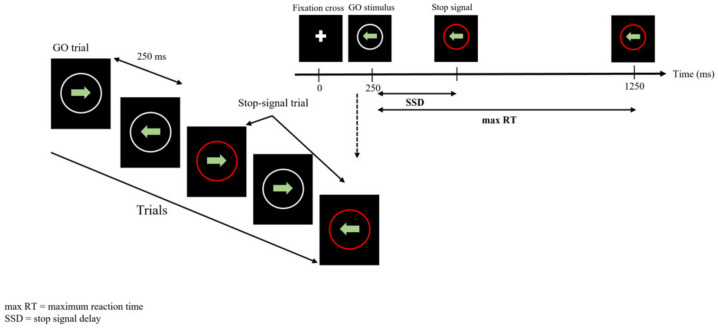
Stop signal Task (SST). Displayed sequences for the SST. In this task, participants responded to a “go” stimulus, and, in a minority of trials (30%), the “go” stimulus is followed by a stop signal that instructs participants to withhold their response.

The stop signal task (SST) is a test of inhibition of prepotent responses. Compared to the go/no-go task (see below) where the no-go sign is presented simultaneously with or instead of the go stimulus, the stop-signal is presented after the go stimulus, so that the response is already in the process of completion. Then, the SST relies on action cancellation, i.e., imposes the suppression of the already initiated motor response [22,23]. Here, the task consisted in a stimulus “green arrow” pointing either to the right or to the left, that appeared at the center of the screen, inviting the participant to hit the corresponding right or left arrow on the keyboard as quickly as possible (Figure 3). In some cases (30%), a red circle appeared around the displayed arrow after a delay varying from 50 to 400 ms (step 50 ms) in an unpredictable manner.

The participants were instructed that the red circle represented a stop signal inviting them to withhold their response [24,25,26]. This task comprised 400 successive stimuli presented in an unpredictable manner to the participant.

In any case, arrows (stimulus) remained displayed for a maximal duration of 1000 ms. The next stimulus appeared 250 ms after either the response of the participant or an elapsed display time.

Inhibition latency associated to the inhibition process engaged in stop signal trials is not measurable directly. This index is computed by estimating the stop signal reaction time (SSRT) from the distribution of reaction times on go-signal only trials [24,26].

### 2.4. Analysis of RR Time Series

A bipolar electrode transmitter belt Polar H10 (Polar, Finland) sampled inter-beat intervals (so-called RR intervals) with an accuracy of 0.001s, based on the electrophysiological signal associated to ventricular contraction (R-peak) transmitted to the surface of the chest. The accuracy in RR time series was demonstrated elsewhere, when comparing the Polar device to classical ECG recordings (e.g., [27,28]).

The RR experimental series comprised 500–700 samples in 10-min baseline recordings, depending on the individual heart rate, and 400–600 samples during ±8 min cognitive tasks, depending on individual heart rates and cumulated response times. The RR time series were imported to Matlab (Matlab_ R2020a, Mathworks, Natick, MA, USA) for subsequent analyses using both Matlab functions and custom-designed algorithms. The RR series were inspected for artifacts. Occasional ectopic beats (irregularity of the heart rate involving extra or skipped heartbeats such as extrasystoles and consecutive compensatory pause) were visually identified and manually replaced with interpolated adjacent values. There were less than 1% corrupted samples in the analyzed series.

#### 2.4.1. Time and Frequency Domains Analyses

The variability in RR time series, the classical heart rate variability (HRV), exhibits interwoven fluctuations covering a large spectrum of magnitude and frequencies. Here, we evaluated the magnitude of variability in HRV, which provides classical markers of tone modulation in autonomic control (sympathetic and vagal), although the present study mainly focused on the temporal structure of the RR time series addressed by entropy and multifractality markers over a number of observational scales. The root mean square of successive differences (RMSSD) in the RR time series was computed. This index relies on the magnitude in short-range (due to differentiation) fluctuations of the heart rate and is generally associated to vagal tone modulation [29]. A quite similar information is generally derived from the power at high-frequencies computed from the power spectral density (PSD) of the RR series. For that, the discrete Fourier transform was computed from 4-Hz resampled RR time series using cubic spline interpolation. The averaged power in predetermined frequency bands were extracted, respectively, at low frequencies (LF) between 0.04 Hz and 0.15 Hz and at high-frequencies between 0.15 Hz and 0.4 Hz, generally associated to a dominantly sympathetic control and a pure vagal control, respectively. The ratio LF/HF power was also assessed, as an indicator of the so-called sympathovagal balance [29].

#### 2.4.2. Entropy in RR Time Series

Complex regulation mechanisms in biological systems have been explored by assessing the absence of obvious regularity in signal output using sample entropy over a range of observational scales [3]. It has been advised that non-stationarity in physiological time series could falsify an entropy-based approach [30,31,32]. On this account, specific procedures were applied in the present work.

To remove unwanted trends, empirical mode decomposition, a data-driven method, was used to decompose the RR series into a sum of intrinsic mode functions and a residual trend. Removing the residual trend avoids errors in entropy calculations due to drift-induced exacerbation of the standard deviation of the series [30]. After that, stationarity was checked using the RWS method [31]. The percentage of stationary signals in each experimental condition can thus be reported.

Refined composite multiscale entropy (RCMSE) based on the algorithm proposed by Wu [11] was computed on detrended series. Using the RCMSE algorithm is particularly advised when applying multiscale entropy methods to short time series. The RCMSE approach addresses most of the issues associated to other multiscale methods [11]. Costa et al. proposed a multiscale entropy (MSE) method consisting of a coarse-graining process and sample entropy computations, to measure complexity at different temporal scales. The coarse-graining process is similar to moving-averaging and the decimation of the original time series, which inevitably shortens the length of the series. As a matter of fact, inadequate (reduced) length in coarse-grained time series may impair accurate computations of SampEn at large scales. Ultimately, in some cases, the SampEn is undefined because no template vectors are matched to one another. Wu proposed CMSE and then RCMSE algorithms that introduced overlapping to improve the number of computer vectors in short series. Yet, compared with MSE and, despite better accuracy, CMSE increases the probability of inducing an undefined entropy in short series. To overcome this issue, RCMSE, while keeping the advantage of CMSE to get a better accuracy when the entropy is defined, adds lower probability of inducing an undefined entropy.

The RCMSE was computed as:

(1) $$RCMSE\left(x,\tau ,m,r\right)=-\ln\left(\frac{\sum_{k=1}^{\tau }n_{k,\tau }^{m+1}}{\sum_{k=1}^{\tau }n_{k,\tau }^{m}}\right),$$

where *x* is the RR intervals time series, *τ* the scale factor, *m* the embedded dimension (here m=2), and *r* the tolerance factor set here to the fixed value of 0.15 [3]. The RCMSE value at a scale factor *τ* is defined as the natural logarithm ratio between the mean of the number of *m* and m+1 the dimensional matched vector pairs $n_{k,\tau }^{m}$ and $n_{k,\tau }^{m+1}$ computed from the *k*-th coarse-grained time series. The proportion of same vectors characterizes the level of signal regularity, an indicator of poor signal complexity. Since signal length uniformization is critical for reliable entropy determination, the length of each analyzed time series was determined based on the shortest series among an individual set of recordings. As a consequence of the series length, RCMSE was assessed over a range of scales from 1 to 4 to preserve >120 samples per scale. An entropy index, Ei, was used to characterize the entropy in a series by a single marker; Ei evaluated the area under the curve of RCMSE vs. scales obtained by using the trapezoidal rule [13,15].

**Entropy: surrogate data testing**

*Shuffled time series*

Background noise has been evidenced in MSE approaches in the sense that white noise exhibits high values of sample entropy at scale 1, although, by definition, white noise exhibits no form of complexity [3]. In white noise, the sample entropy drops markedly in larger scales, which is not the case if critical information exists in the signal over a range of scales, as in the case of biological signals. To distinguish white noise from complex RR time series, each experimental series was shuffled (n=50 shuffle surrogates) and RCMSE was calculated for each participant in each cognitive task. A distinct profile between experimental and surrogate series was a prerequisite for considering that the entropy in our RR time series is not the consequence of background noise (see Figure 10 in Results).

*Phase randomized time series*

In addition, we generated 50 phase-randomized surrogate series with a Fourier-phase randomizing algorithm, a method called Iterative Amplitude Adjusted Fourier Transformed IAAFT [33]. Since the FT method distorts the amplitude distribution of the original series when the sample distribution is not Gaussian, the IAAFT method implemented an iterative procedure (here the number of iterations was 200) that alternatively constrains the surrogate series to have the same power spectrum and the same amplitude distribution (by a rank-ordering procedure) of the experimental series. In agreement with recent analyses focusing on methodological bias with short-term HRV series [34,35], the IAAFT analysis allowed operating detection and quantification of nonlinear components in our RR time series. At the individual level, detection consisted in observing if the experimental Ei values computed with RCMSE fall below the 2.5th percentile of the Ei computed over the IAAFT surrogate series [35]. Then, for each situation, the number of participants exhibiting an RR series with Ei values below the 2.5th percentile was quantified (see Figure 11 in Results).

**Additional analyses**

*Linear MSE*

In order to provide additional support to the results obtained by applying RCMSE to RR time series, we also computed linear MSE (LMSE). LMSE is based on linear state-space models to assess the complexity of a process [12]. As RCMSE, LMSE has been recommended for entropy measurement in short time series, especially when nonlinear features are suspected. Linear sample entropy at a given scale $\tau_{s}$ is obtained as:

(2) $$LSE_{x}\left(\tau_{s}\right)=\frac{1}{2}ln2\pi e\frac{\sigma_{ed}^{2}}{\sigma_{xd}^{2}},$$

where x represents here the RR time series, $\sigma_{x_{d}}^{2}$ and $\sigma_{e_{d}}^{2}$ are, respectively, the variance of the down-sampled process $x_{d}$ and that of the relevant innovations $e_{d}$. Following the same procedure as that used for RCMSE, an index of entropy was obtained by computing the integral (trapezoidal rule) of the LSE values over scales 1 to 4.

*Conditional entropy*

In addition to multiscale computations, we calculated the conditional entropy using model-free estimators to evaluate how estimators could provide convergent results and strengthen the conclusions about task-specific complexity in beat-to-beat dynamics. We estimated the conditional entropy with model-free estimators based on binning (Bi) [36], on kernel (Ke) [37] and on nearest neighbors (NN) [37,38].

#### 2.4.3. Multifractal Properties of RR Time Series

**Figure 4:**
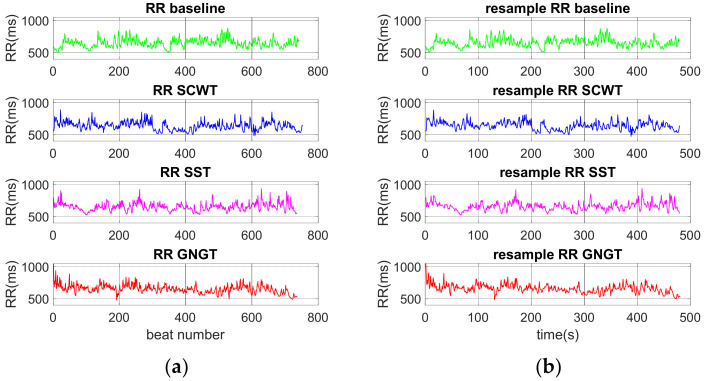
Typical RR time series for one subject in each phase (baseline, SCWT, SST, GNGT). (**a**) Original RR time series, (**b**) 1-Hz resampled RR series.

In physiology, the term fractal was used to designate self-similarities in biological times series. Detrended fluctuation analysis (DFA) is a popular method for assessing self-similarity in biological signals that provides a coefficient *α* strictly related to the Hurst’s exponent [39]. Recent advances based on DFA make it possible to evaluate multifractal coefficients over a range of observational scales, which represent a finer analysis to explore the scale-free behavior in biomedical signals [16,17]. As RR time series are inherently irregularly sampled, there is a need to transform an observational scale (a number of samples) into a time scale [16,17]. To get the best matching in the present study, the RR time series were resampled at 1Hz before running MM-DFA. Figure 4 shows high a similarity in the original and resampled series.

Here, for each 1Hz-resampled RR time series, the self-similarity scaling exponent was calculated as a function of scale *n* and of the multifractal order *q* applying the multifractal multiscale DFA based on works by Castiglioni et al. [16,40]. In short, the RR time series are first integrated by cumulative summation after mean subtraction.

(3) $$y\left(k\right)=\sum_{j=1}^{i}x\left(\left(j\right)-\bar{x}\right),$$

The cumulative sum is split into M blocks of size *n* using maximally overlapped blocks. The variance of the residuals in the *k*-th block (1 ≤ *k* ≤ M) after detrending with a least-square detrending first-order polynomial *p*(*i*) is:

(4) $$\sigma_{n}^{2}\left(k\right)=\frac{1}{2}\sum {\left(y\left(i\right)-p\left(i\right)\right)}^{2},$$

so that the DFA variability function is:

(5) $$Fq\left(n\right)={\left(\frac{1}{M}\sum_{k=1}^{M}{\left(\sigma_{n}^{2}\left(k\right)\right)}^{\frac{q}{2}}\right)}^{\frac{1}{q}}\;for\;q\neq 0,$$

(6) $$Fq\left(n\right)=e^{\frac{1}{2M}\sum_{k=1}^{M}\ln\left(\sigma_{n}^{2}\left(k\right)\right)}\;for\;q=0,$$

**Figure 5:**
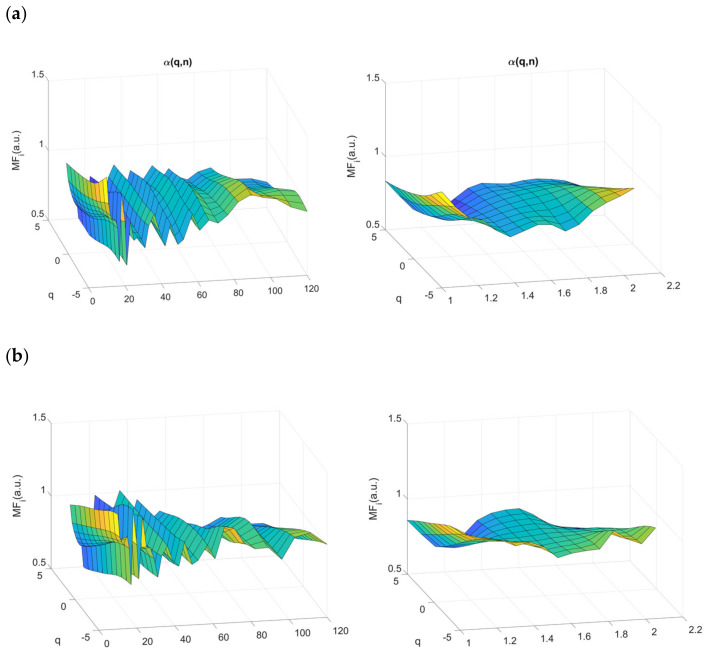

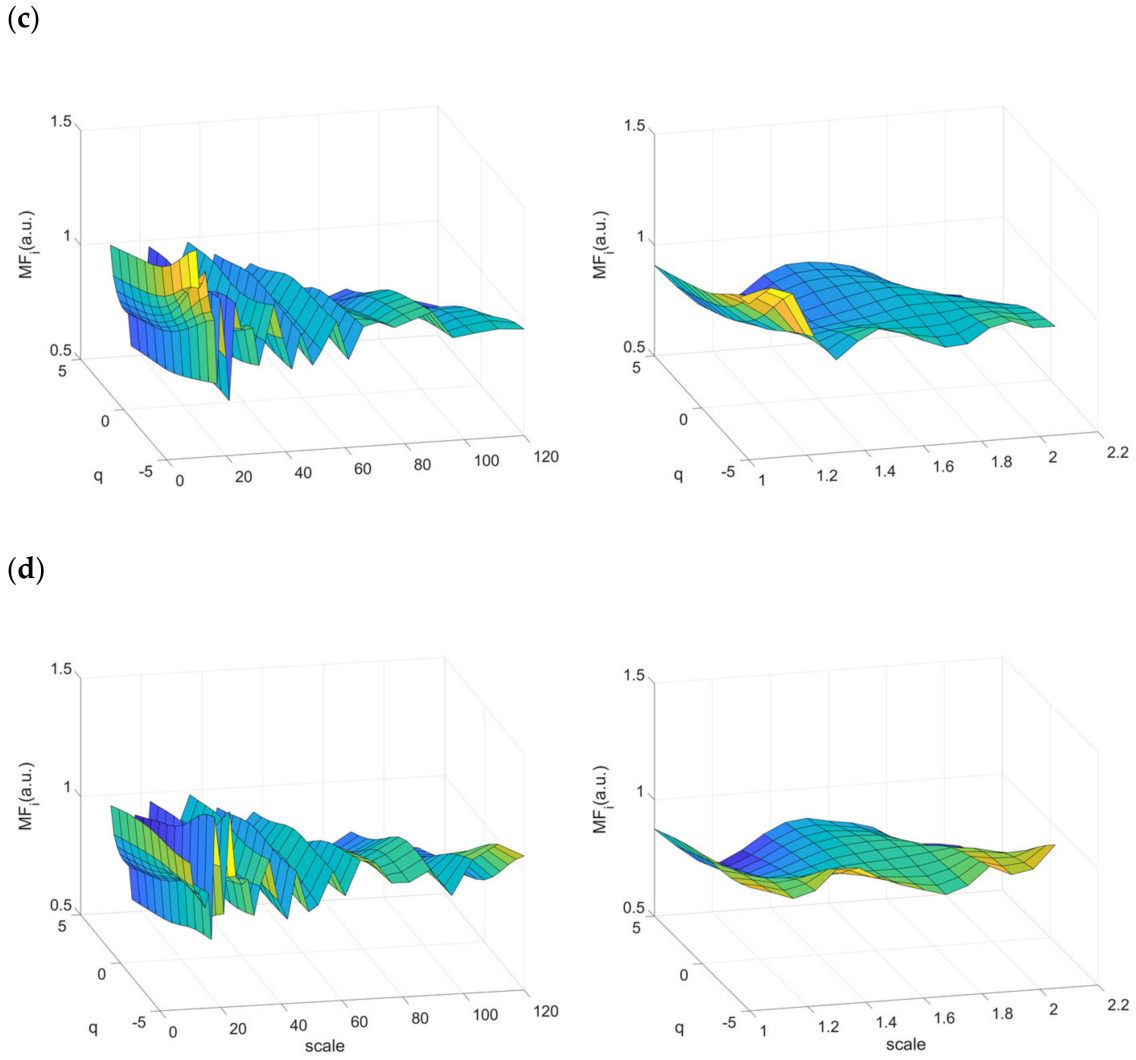
Surfaces of multifractal multiscale DFA average over all participants obtained on each condition (baseline (**a**), SCWT (**b**), SST (**c**) and GNGT (**d**)). Surface computed with the method described in [16] on the left side or with the fast one described in [40] on the right side.

**Figure 6:**
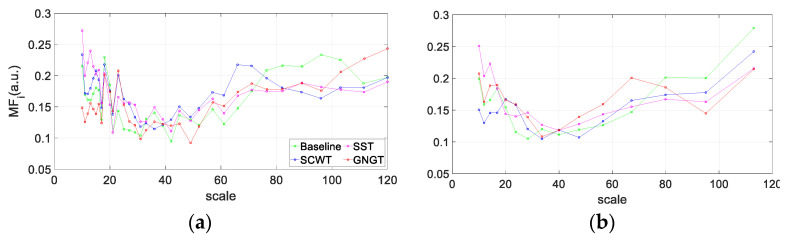
Multifractal index (MFi) computed as a function of observational scales *n* with the method described in [16] (**a**) or with the fast one described in [40] (**b**). Computation for each experimental situation, baseline, SCWT, SST and GNGT.

The variability function is calculated for block sizes *n* between 10 and 128. The scaling exponent is the local slope around the scale *n* of log F*q*(*n*) vs. log *n*. The method used in the present study to get the local slopes deserves further details since we used both the 5-point derivative used in [16] (Equation (5) and related equations for extrema) and in [40] (Equation (22)). The difference is that the exact equispaced log *n* values in the latter case are calculated by spline cubic interpolation of the log F*q*(*n*) samples. The reason why the increments are not exactly evenly spaced lies in the fact that *n* is an integer, a rounded value, so that successive increments due to the residual error are underestimated then overestimated and conversely. The result is a “zig-zag” pattern as illustrated in Figure 5 and Figure 6. Interpolation provides a smoother profile. To shift away from potential “methodological noise” in our analyses, the results obtained with each method are provided. Examples of multifractal and multiscale coefficients (*q*,*n*) obtained with both methods of local slope calculation are shown in Figure 5.

**Figure 7:**
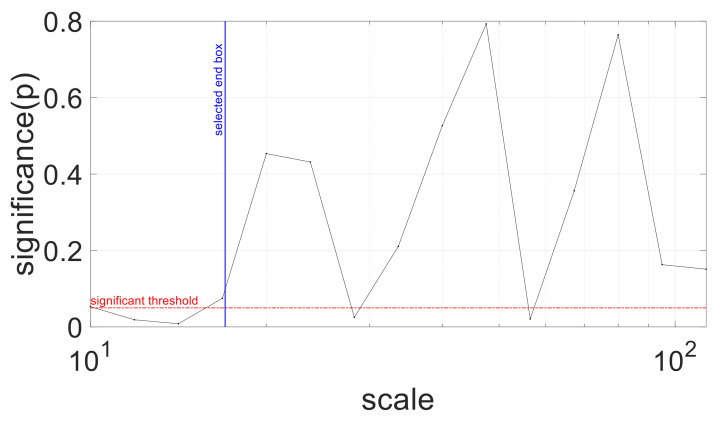
Significance of repeated measures ANOVA conducted on MFi parameter at each observational scale. Blue line indicates the end of the range of scales where multifractality differed according to the situation, between n=10s and n=17s.

In order to define a multifractal index as a function of scale *n*, MFi(*n*) was obtained by calculating the standard deviation of all *α*(*q,n*). In Figure 6, MFi was plotted as a function of observational scales *n*. To compare the cognitive tasks, we finally obtained a concise multifractal index called MFI by calculating the area under the curve (trapezoid rule) obtained with the fast MM-DFA computation method [40] (Figure 6b) between scale n=10 s and scale n=17 s, because within this particular range of scales repeated measures ANOVA showed significant difference among the baseline, SCWT, SST and GNGT (Figure 7).

**Multifractality: surrogate data testing**

**Figure 8:**
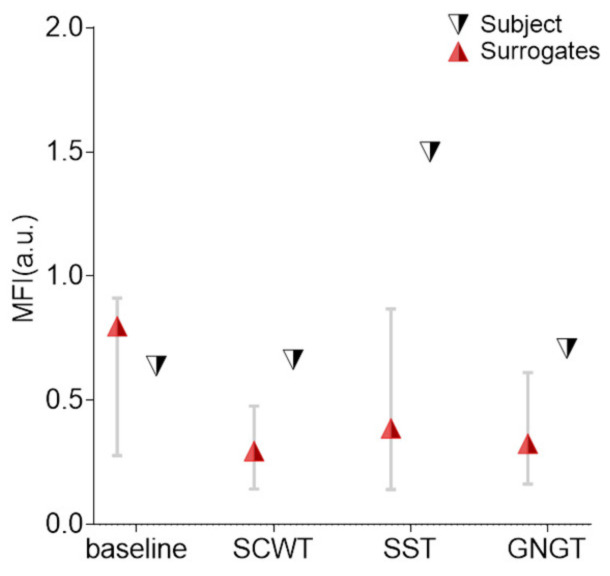
Values of the nonlinear dynamic measures on RR time series for a typical subject, obtained on each experimental condition (baseline, SCWT, SST and GNGT). The markers are the original values and the errors bars are the 5th and 95th percentiles of the surrogates’ distribution.

From the 1-Hz resampled experimental RR time series, we generated 50 surrogate series using IAAFT. We operated the so-called detection and quantification of nonlinear components in our multifractal RR time series. The detection consisted in observing if the experimental MFi values considering MFI computation scales lies within or outside the 95% confidence interval based on the percentile of the distribution among surrogates (Figure 8). Then, for each situation, the number of participants exhibiting RR series with MFi values outside the 95% confidence interval was quantified (see Figure 13 in Results).

### 2.5. Statistical Analysis

Results were tested for normality (Shapiro–Wilk test) and homogeneity (Levene test). The values are expressed as mean and standard deviation to the mean (SD). Outliers were identified using inter quartile range method described by Tukey in 1977 and the values of outliers were replaced by the mean value for the considered sample.

To test task effects on heart rate dynamics, we used both standard statistical tests and Bayesian equivalents to extend insight and guide interpretation of the significance (*p* values), according to the likelihood of the alternative hypothesis versus the null hypothesis [41,42]. Indeed, a disadvantage of null hypothesis significance testing is that nonsignificant *p* values (e.g., when reporting no condition effect on experimental measures) cannot be interpreted as support for the null hypothesis [42,43].To circumvent this issue and confirm whether the potential non-significant findings reported potential support for the null hypothesis, we calculated the Bayes factor (BF): specifically, we computed the log scale of BF10 (Bayes factor giving evidence for the alternative hypothesis H_1_ over the null hypothesis H_0_) noted log(BF10) that can be easily interpreted such that a negative value indicates support for the null hypothesis, whereas a positive value indicates evidence in favor of the alternative hypothesis (see Supplementary Materials Table S2 for an interpretation scale of log(BF10)) [44].

For standard analyses, we conducted repeated measures ANOVA or the nonparametric equivalent, the Friedmann test. For standard post-hoc tests, we applied the Holm correction for multiple comparisons. For the Bayesian analyses, we used the default JASP priors (paired sample t-tests: medium effect size on a Cauchy distribution of 0.707; repeated measures ANOVA: r-scale fixed effects of 0.5, r-scale random effects of 1, and r-scale covariates of 0.354), and our model was compared to the null model for Bayesian repeated measures ANOVA [43]. Standard and Bayesian analyses were performed with the task (task effect) as a within-participants factor.

Receiver Operating Characteristic (ROC) curves were used to measure the sensitivity and specificity of the indices computed [45]. The area under the ROC curve (AUC) was computed for each index. This area represents the accuracy of the index in a binary classification paradigm [46]. Yi corresponds to a measure of a diagnostic test’s ability to balance the sensitivity and specificity (Yi=sensitivity+specificity − 1).

Data were analyzed in JASP (version 0.14.1, https://jasp-stats.org/, accessed on 20 May 2021).

## 3. Results

### 3.1. Cognitive Performance: Response Time and Accuracy

A set of metrics including individual response time and accuracy served for quantifying cognitive tasks performance was obtained (Table 1). The highest response time (RT) was observed in SCWT and amounted to 879 ± 119 ms. SCWT imposes a trade-off between speed and response accuracy. The small variability in response time may rely on the instructions given to the participants in our conditions, encouraging accuracy rather than speed.

The response time in SST when compared to GNGT is informative about the time needed to initiate and then bias the sensorimotor response in SST but not in GNGT. Thus, as expected, a greater response time was observed in SST, 496 ± 69 ms, when compared to GNGT, 405 ± 64 ms (*p* < 0.001, log(BF_10_) = 18.3).

### 3.2. Classical Metrics in RR Time Series

Heart rate dynamics were described from classical time-domain and frequency-domains autonomic markers but, more importantly, regarding our hypotheses by using markers of signal complexity.

Descriptive statistics of the RR time series are summarized in Table 2. The absence of change in the average RR interval duration (between SST and GNGT vs. baseline) indicates that the heart rate per se was not adequate as a task-specific marker, which also holds for autonomic markers, RMSSD, LF, HF and LF/HF (Supplementary Materials Table S1). In addition, classical autonomic markers demonstrated poor sensitivity as quantified by the ROC curves analysis. They never reached the highest AUC or Youden’s index (Yi) value, except for a marginal advantage for the AUC of LF in the situation of GNGT (Table 4).

### 3.3. Multiscale Entropy in RR Time Series

**Figure 9:**
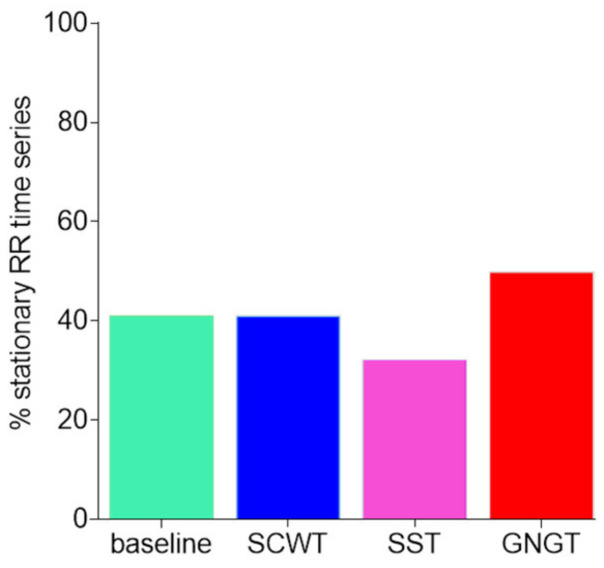
Percentage of stationary signals obtained by counting the subjects. Stationarity was tested using the RWS method. Results are presented for each experimental condition, baseline, Stroop color and word task (SCWT), stop signal task (SST) and go/no-go task (GNGT).

As introduced in Methods, given that non-stationarity might affect the entropy measurement, an RWS test was conducted on RR time series. The percentage of stationary time series in our conditions reached about 40% (Figure 9). More importantly, this percentage was not significantly different across the experimental conditions (p=0.49), thus indicating that differences in the task-specific entropy can hardly result from a bias due to a singularity in non-stationarity.

**Figure 10:**
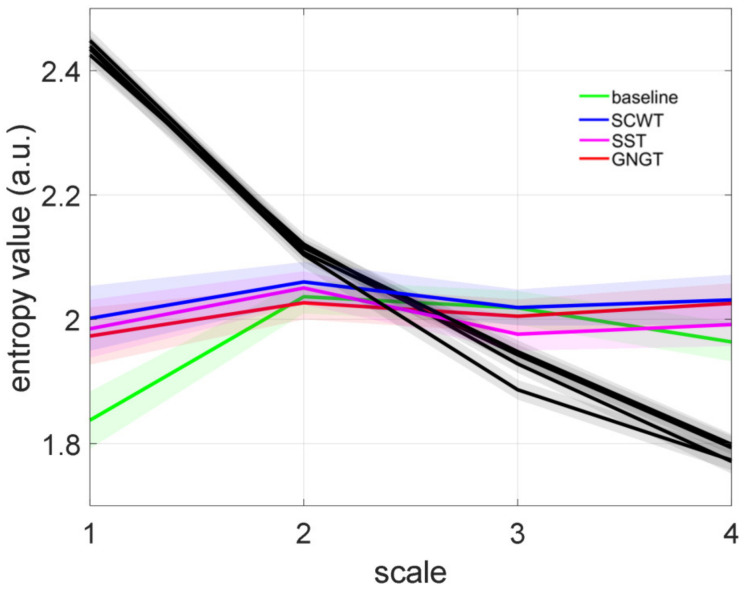
Refined composite multiscale entropy (RCMSE) analysis of RR interval time series. Sample entropy values at time scales 1 to 4 during all conditions (baseline, Stroop color and word task (SCWT), stop signal task (SST) and go/no-go task (GNGT) (mean ± SD)). The RCMSE curves for the surrogate shuffled time series (black curves) are also presented. The entropy index Ei represents the trapezoid approximation of the area under each curve.

As explained in Methods, entropy vs. scales computed from RCMSE in experimental times series was compared to entropy vs. scales in shuffled series. Figure 10 indicates that the shuffled surrogates showed the typical profile of a white noise, as expected. That was clearly different from the entropy profiles in the experimental series.

**Figure 11:**
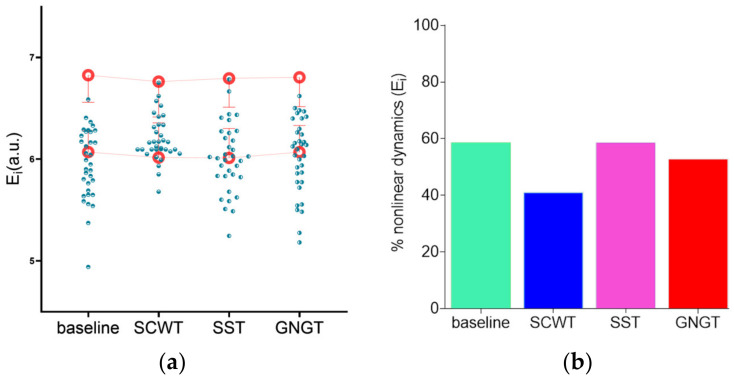
(**a**) Surrogate data testing for entropy measurement. Open red circles represent the mean entropy value and standard deviation of the 97.5th and 2.5th percentiles from 50 surrogate series, green circles represent subjects’ individual values. (**b**) The percentage of significant nonlinear dynamics obtained by counting the subjects. For these subjects, the original RR time series presented an entropy value below the 2.5th percentile of 50 surrogates’ values. Results are presented for each experimental condition, baseline, Stroop color and word task (SCWT), stop signal task (SST) and go/no-go task (GNGT).

As for IAAFT (phase-randomized) surrogate testing, Figure 11b illustrates the percentage of the subjects who demonstrated nonlinearities (Figure 11a), which amounted to 40 to 60% of the participants in our conditions.

**Figure 12:**
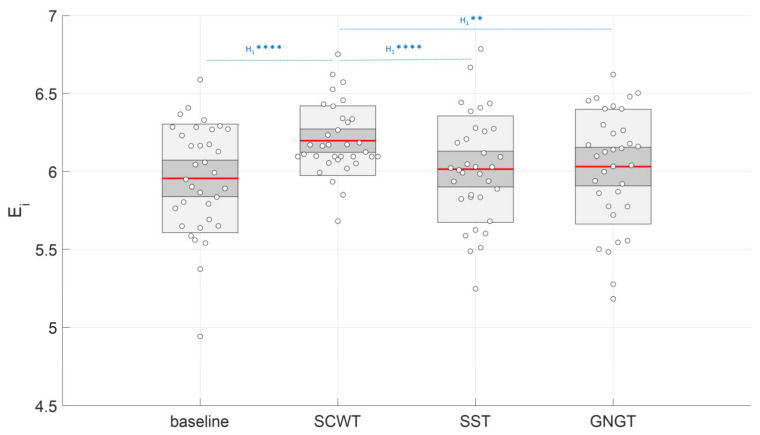
Effect of cognitive tasks (SCWT = Stroop color and word task; SST = stop signal task and GNGT = go/no-go task) on the entropy index (Ei) value. a.u. = arbitrary unit. Interpretation scale: H_1_ **** and H_1_ ** mean, respectively, extreme and strong evidence for the alternative hypothesis.

By contrast with the results obtained with classical markers, the entropy index obtained from the area under the curve, Ei varied with task as shown by the repeated measurements ANOVA (*F*_(3,99)_ = 4.67, *p* < 0.01, log(BF_10_) = 2.20). The post-hoc analysis showed that E_i_ reached a higher value during the SCWT (Supplementary Materials Table S1, Figure 12) when compared to the baseline (*p* < 0.01, log(BF_10_) = 2.76), to SST (*p* < 0.05, log(BF_10_) = 2.29) and to GNGT regarding Bayesian analysis (*p* = 0.06, log(BF_10_) = 1.43) (Figure 12). In other words, the entropy in RR time series specifically increased in the inhibition test that involved cognitive interference, SCWT. In agreement, Ei was the most sensitive marker allowing to distinguish SWCT from the baseline (Figure 15a and Table 4).

**Additional analyses**

Descriptive statistic results of additional entropy analyses are presented in Table 3. Among the four measurement methods, two of them, LMSE and the conditional entropy computation using the kernel estimator, show similar dynamics to those observed with RCMSE. For these two methods, we observe the highest entropy values in the SCWT.

### 3.4. Multifractality in RR Time Series

**Detection of nonlinearities**

**Figure 13:**
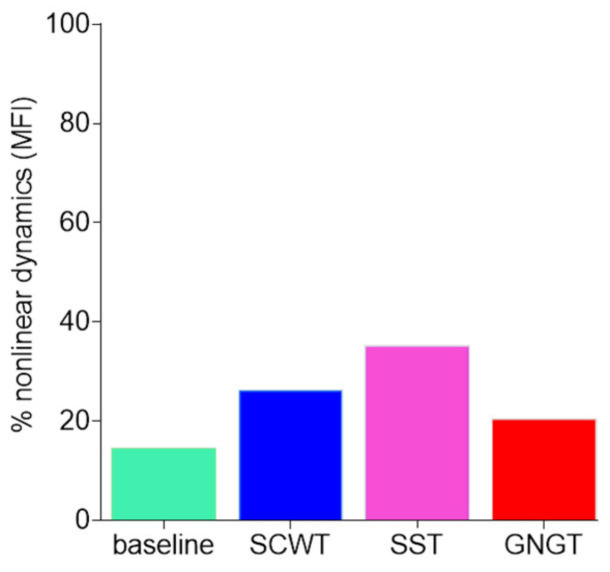
Percentage of significant nonlinear dynamics obtained by counting the subjects. For these subjects, the original RR time series presented more multifractal dynamics than the surrogate ones. Results are presented for each experimental condition, baseline, Stroop color and word task (SCWT), stop signal task (SST) and go/no-go task (GNGT).

Figure 13 shows the percentage of subjects who demonstrated nonlinearities in multifractal RR dynamics based on the phase-randomization surrogate data testing. Nonlinearities were shown in the range of 20 to 35% of the participants, in each cognitive task, while it amounted to only 14% in the baseline task.

**Quantification of scale-specific multifractality**

**Figure 14:**
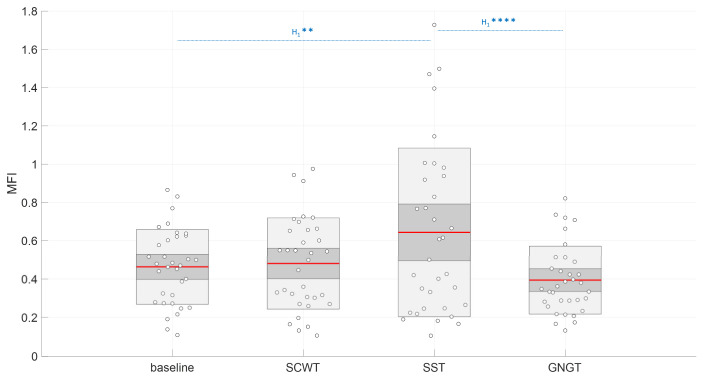
Effect of cognitive tasks (SCWT = Stroop color and word task; SST = stop signal task and GNGT = go/no-go task) on multifractality index (MFI) value. a.u. = arbitrary unit. Interpretation scale: H_1_ **** and H_1_ ** mean, respectively, extreme and strong evidence for the alternative hypothesis.

The multifractality index MFI obtained from the area under the curve of MFi vs. scales 10-to-17 was significantly different among the experimental situations (*F*_(3,99)_ = 11.4, *p* < 0.01, log(BF_10_) = 3.71). The post-hoc analysis showed significantly higher MFI in SST when compared to baseline, indicated by Bayesian analysis (*p* = 0.08, log(BF_10_) = 1.03) and when compared to GNGT (*p* < 0.01, log(BF_10_) = 3.46) (Supplementary Materials Table S1, Figure 14).

### 3.5. Sensitivity Analysis: ROC Curves

**Figure 15:**
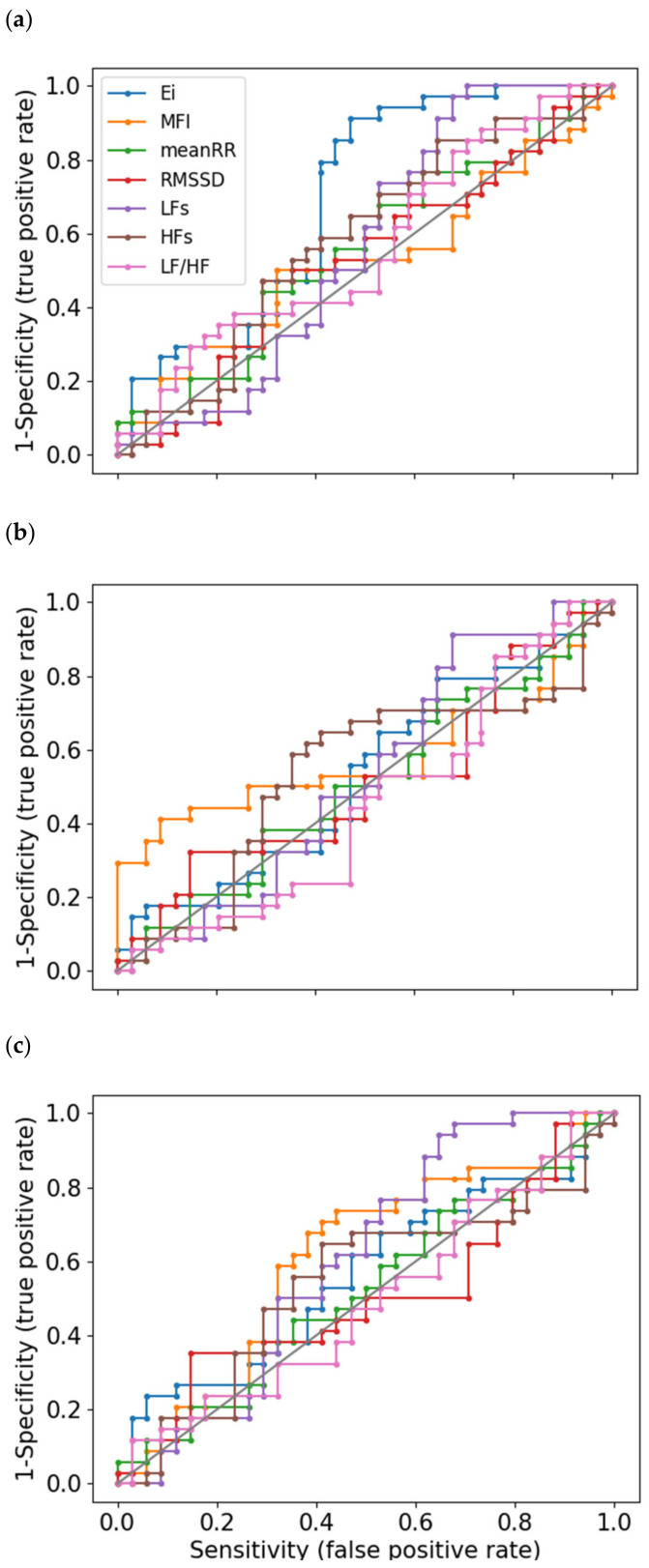
Receiver operating characteristic (ROC) curves (sensitivity vs. 1-specificity) for RR interval time series indices. Binary classification between Stroop color and word task (SCWT) and baseline (**a**), stop signal task (SST) and baseline (**b**) and go/no-go task (GNGT) and baseline (**c**).

The ROC curves analysis including all measured heart rate metrics is presented in Figure 15. The corresponding area under the curve (AUC) and Youden’s indices (Yi) are reported in Table 4. In each of the three tasks (SCWT, SST, GNGT), the greatest AUC were obtained either in the entropy or multifractality indices. This demonstrates how these indices outperformed the classical cardiac autonomic markers. This was mainly true for the entropy in SCWT (Figure 15a and Table 4).

## 4. Discussion

Based on cognitive-autonomic interactions and the resulting interdependencies across brain-to-heart networks governed by nonlinear processes, the present analysis of complexity in RR time series during specific cognitive tasks provides three main findings, in agreement with the initial hypotheses: (i) nonlinear metrics, here the entropy and multiscale-multifractality best distinguish specific cognitive-autonomic architectures associated to the selected task; (ii) despite that response inhibition was a common construct in every task, the entropy, a known marker of complex interactions [13], increased specifically in the presence of cognitive interference (Stroop color and word task, SCWT); (iii) otherwise, the multifractality, a marker of effective adaptation associated with a greater number of interacting brain networks [18,47], increased at several scales, specifically during the action cancellation task, known to need biasing an initially recruited sensorimotor network. Although this study was not designed to provide direct evidence of brain network organization, the intuition that specific cognitive-autonomic interdependencies could be inferred from nonlinear behavior beyond the confines of the brain, seems to be in good agreement with our main findings based on cardiac heart rate dynamics.

### 4.1. A Cognitive Architecture Reflected in HRV Time Series

Although cognitive tasking sounds like a generic way to signify that specific processes have been stimulated by a number of mental stimulations, researchers have been able to introduce specific task structuration that triggers different neural dynamics for years. Even similar cognitive tasks involving response inhibition, which refers to the suppression of prepared or initiated actions, that have been sometimes used interchangeably to capture individual profiles, have been shown to introduce different hierarchical coordination and fine specificity in neural dynamics [22,23]. In the present work, we posit that these very neural dynamics may have direct consequences on the temporal structure of heart rate time series, given interdependencies between the cognitive and the autonomic circuitry [48].

### 4.2. Specificity of the Nonlinear Metrics

The critical intuition in elaborating our analysis was that under the interaction-dominant hypothesis, the phenomenon of cognitive functions is an emergent property that arises from system networks interactions [49,50,51]. Cognition is embodied in dynamically intricate networks where nonlinear processes are critical for the emergent control architecture, wherein the information flow spans several temporal scales. Admitting that task-specific neural dynamics and network complexity go hand in hand, one could conceive that nonlinear metrics could be well indicated to account for subtle coordination among cognitive-autonomic interactions. Critically, in recent years, just such metrics of heart rate variability have shown a finer interpretation of brain–heart interactions [9,10,13,14].

In this vein and as a critical outcome in the present work, entropy and multifractal metrics outperformed classical markers of cardiac autonomic modulations computed in the time- and frequency-domain, in distinguishing individual responses to selected cognitive tasks (Table 4). In other words, entropy and multifractality are able to report on the specificity of the cognitive functioning in SCWT and in SST, respectively, whereas markers of autonomic arousals are not. How entropy and multifractality provide insight into the complex neural organization and hierarchical coordination in cognitive functions deserves a more detailed discussion.

### 4.3. Improved Entropy in SCWT

As illustrated in Figure 12, our study points to a singular response in entropy (Ei) in SCWT. The Stroop color and word task requires resolving cognitive interference while the global response is also influenced by recent trial history as reflected in sequence effects [52]. Neuroimaging investigations have associated the causal involvement of the prefrontal cortex and point to critical neural dynamics in that structure for adaptive cognitive control [53]. Emerging control is implemented after receiving input from the anterior cingulate cortex suggesting that the network between the prefrontal cortex and the anterior cingulate cortex can flexibly maintain or reset previously experienced signals in the service of successful performance [52]. Interestingly, these cortical structures are common to the central autonomic network (CAN) described by Benarroch [5] and Thayer [6,54]. Our group provided recent suggestions that the functional organization of the CAN is reflected in heartbeat dynamics [6]. It is confirmed in the present study that RCMSE could capture the main features in cognitive interactions. Nevertheless, as shown by the slightly different results obtained by using LMSE to distinguish SCWT from the other tasks, a fine analysis of subtle interactions should rely on using different complementary approaches of entropy. Our main hypothesis here—a causal link between a specific cognitive-autonomic architecture and nonlinear dynamics in RR time series—seems to be verified by the specific improvement in entropy when the response inhibition meets the cognitive interference during SCWT. This result brings about a new facet of entropy in peripheral autonomic control as a reliable marker to capture individual cognitive structures beyond the confines of the brain.

### 4.4. Multifractality in SST

Inhibitory control is generally referred to as multi-network executive function that is critical for flexible responsiveness to changing environmental demands. Cognitive tasks known as the stop signal task (SST) and go/no-go task (GNGT) have been designed to capture individual performances in response inhibition. First, they distinguish themselves from SCWT by the absence of cognitive interference. In our experimental context, this is an argument to explain different signatures reflected in entropy as discussed above. Second, the execution of SST and GNGT relies on cascades of neural processes that differ in terms of a “simple” action restraint dominating in GNGT, but a more “complicated” action cancellation—after having engaged sensorimotor neural dynamics—that dominates in SST [23]. The later scenario requires that participants repeatedly adapt a cognitive architecture that engages specific brain networks associated to sensorimotor processes. A critical finding here was the putative multifractal signature of these specific cognitive-autonomic interdependencies, reflected in heart rate time series. Recently, the degree of multifractality in output signal of a sensorimotor system has been shown a marker of effective adaptation [18], reflecting a greater number of interacting brain networks [47]. In agreement, an interpretative hypothesis here is that the richer coordinated network operating in SST due to the prepotent motor response and intentional biasing might be the cause of greater cognitive-autonomic multifractality reflected in peripheral cardiac autonomic control. Without additional direct measurements of the brain network connectivity, this suggestion remains yet speculative.

## 5. Conclusions

How task-specific cognitive architectures influence the dynamic coordination of brain-to-heart networks seems to be reflected beyond the confines of the brain, in nonlinear dynamics of the heart rate. This has interesting consequences in human monitoring situations where direct brain measurements can hardly be conducted. Our results suggest that the reliability of entropy markers could be taken further to reveal ad hoc adaptations that concern specific networks. In addition, how the cognitive-autonomic network aggregate parallel network activities, e.g., a sensorimotor inhibition, could be finely captured thanks to recent developments of a multifractal-multiscale analysis. Although the present study is grounded on interpretative hypotheses, the potential of entropy and multifractality metrics in biosignals to capture fundamental behavior in complex networks is valued here.

## Figures and Tables

**Table 1:** Descriptive statistics (mean ± standard deviation) of indices computed from cognitive tasks.

Variable (Unit)	SCWT	SST	GNGT
**RT** (ms)	879 ± 119 ^c^	496 ± 69 ^c^	405 ± 64 *
**perf** (%)	97.0 ± 2.25	99.3 ± 0.83	97.6 ± 1.80
**SSRT** (ms)		308 ± 32.4	
**inhib perf** (%)		60.4 ± 22.7	

RT = response time; perf = percentage of correct responses (note that for SST, “perf” represents the percentage of responses for which a subject responds correctly when only a GO stimulus is presented); SSRT = stop signal reaction time; inhib perf = inhibition performance in SST (percentage of trials where a subject correctly withholds his response when a stop signal followed a GO stimulus); SCWT = Stroop color and word task; GNGT = go/no-go task; SST = stop signal task. * indicates significant difference with (^c^) compared task.

**Table 2:** Descriptive statistics (mean ± standard deviation) of indices computed from RR time series analyses.

Variable (Unit)	Baseline	SCWT	*SST*	GNGT
**meanRR** (ms)	841 ± 131^c^	812 ± 131 *	836 ± 131 ^c^	832 ± 135 ^c^
**RMSSD** (ms)	43.3 ± 18.9	42.0 ± 18.1	45.3 ± 21.1	44.7 ± 21.7
**LF** (ms^2/Hz)	1471 ± 927	1165 ± 462	1291 ± 643	1064 ± 463
**HF** (ms^2/Hz)	886 ± 630	685 ± 515	862 ± 777	862 ± 761
**LF/HF**	2.25 ± 1.69	2.62 ± 1.95	2.23 ± 1.19	2.23 ±1.51
**E_i_** (a.u.)	5.96 ± 0.35 ^c^	6.20 ± 0.22 *	6.01 ± 0.34 ^c^	6.03 ± 0.37 ^c^
**MFI** (a.u.)	0.46 ± 0.20 ^c^	0.48 ± 0.24	0.64 ± 0.44 *	0.39- ± 0.18 ^c^

meanRR = mean interbeat interval duration; RMSSD = root mean square of successive differences; LF = low frequency power in HRV power spectrum; HF = high frequencies power in HRV power spectrum; LF/HF = ratio of low frequencies on high frequencies; E_i_ = entropy index; MFI = multifractal index. * indicates significant differences with (^c^) compared conditions. SCWT = Stroop color and word task; GNGT = go/no-go task; SST = stop signal task.

**Table 3:** Descriptive statistics (mean ± standard deviation) of the four entropy indices computed from RR time series in additional analyses.

Entropy Indices (Unit)	Baseline	SCWT	*SST*	GNGT
**LMSE** (a.u.)	2.90 ± 0.52	3.19 ± 0.44	3.09 ± 0.52	3.09 ± 0.48
**CEBi** (a.u.)	1.02 ± 0.11	1.00 ± 0.16	1.03 ± 0.15	1.01 ± 0.16
**CEKe** (a.u.)	1.84 ± 0.27	2.00 ± 0.30	1.98 ± 0.27	1.98 ± 0.20
**CENN** (a.u.)	5.31 ± 0.30	5.27 ± 0.27	5.34 ± 0.28	5.25 ± 0.35

LMSE = linear multiscale entropy; CEBi = conditional entropy computed with binning model-free estimator; CEKe = conditional entropy computed with the kernel model-free estimator; CENN = conditional entropy computed with the nearest neighbor model-free estimator. SCWT = Stroop color and word task; GNGT = go/no-go task; SST = stop signal task.

**Table 4:** Sensitivity analysis (area under the ROC curve (AUC) and Youden’s index (Yi)).

Indices	SCWT/Baseline		SST/Baseline		GNGT/Baseline	
	*AUC*	*Y_i_*	*AUC*	*Y_i_*	*AUC*	*Y_i_*
**meanRR**	0.55	0.15	0.51	0.09	0.52	0.08
**RMSSD**	0.53	0.18	0.51	0.18	0.50	0.21
**LF**	0.56	0.29	0.53	0.24	0.60	0.29
**HF**	0.58	0.21	0.53	0.24	0.54	0.24
**LF/HF**	0.56	0.15	0.45	0.08	0.49	0.08
**E_i_**	0.69	0.44	0.53	0.15	0.56	0.18
**MFI**	0.52	0.18	0.58	0.32	0.61	0.29

meanRR = mean interbeat interval duration; RMSSD = root mean square of successive difference; LF = low frequency power in HRV power spectrum; HF = high frequencies power in HRV power spectrum; LF/HF = ratio of low frequencies on high frequencies; E_i_ = entropy index; MFI = multifractal index. SCWT = Stroop color and word task; GNGT = go/no-go task; SST = stop signal task.

## Data Availability

The datasets generated and analyzed during the current study are available from the corresponding author on reasonable request.

## Supplementary Materials

The following are available online at https://www.mdpi.com/article/10.3390/e23060663/s1, Table S1: Task effect on HRV indices (standard statistics and Bayesian equivalents are reported), Table S2: Interpretation scale of log (BF_10_) factor based on Jeffreys 1961.
