# Supplementary material for: Entropy and Multifractal-Multiscale Indices of Heart Rate Time Series to Evaluate Intricate Cognitive-Autonomic Interactions
Source: Entropy (Basel). 2021 May 25;23(6):663. doi: 10.3390/e23060663 (PMC8230296; doi:10.3390/e23060663)
Supplement: Supplementary file 1 [file entropy-23-00663-s001.zip › entropy-1223102-supplementary.pdf]

Supplementary Materials

Table 1. Task effect on HRV indices (standard statistics and Bayesian equivalents are reported).

**Statistic test**	**Features**											
	**meanRR***			**RMSSD***			**LF***			HF*		
	F	***p.value***	***log(BF₁₀)***	***F***	***p.value***	***log(BF₁₀)***	***F***	*p.value*	*log(BF₁₀)*	*F*	*p.value*	*log(BF₁₀)*
**ANOVA**	0.96	<0.001	2.33	0.93	0.75	−1.52	0.64	0.26	1.60	0.83	0.29	−0.93
**Post-hoc tests**												
**baseline/SCWT**	3.89	<0.001	1.28	0.61	1.00	−1.47	0.52	1	0.52	1.73	0.516	0.06
**baseline/SST**	0.75	0.98	−1.50	0.47	1.00	−1.09	0	1	−1.12	0.75	1	−1.66
**baseline/GNGT**	0.98	0.98	−0.97	0.14	1.00	−1.33	1.73	0.516	1.71	0.14	1	−1.65
**SCWT / SST**	3.14	<0.05	2.74	1.08	1.00	−0.33	0.52	1	−0.88	0.98	1	−0.39
**SCWT / GNGT**	2.91	<0.05	1.98	0.75	1.00	0.08	1.22	0.904	−0.85	1.59	0.571	0.22
**SST / GNGT**	0.23	0.98	−1.52	0.33	1.00	−1.64	1.73	0.516	0.99	0.61	1	−1.69

	LF/HF*			MFI*			E_i		
	F	*p.value*	*log(BF₁₀)*	*F*	*p.value*	*log(BF₁₀)*	*F*	*p.value*	*log(BF₁₀)*
**ANOVA**	0.72	0.16	−2.02	0.46	<0.01	2.14	4.67	<0.01	2.19
**Post-hoc tests**									
**baseline/SCWT**	1.92	0.35	−1.18	0.61	0.86	−1.63	−3.56	<0.01	2.76
**baseline/SST**	1.50	0.57	−1.69	2.44	0.08	1.03	−0.87	0.81	−1.41
**baseline/GNGT**	0.33	1	−1.69	0.80	0.86	−0.40	−1.11	0.81	−1.24
**SCWT / SST**	0.42	1	−0.79	1.83	0.28	0.08	2.69	<0.05	2.29
**SCWT / GNGT**	1.59	0.57	−0.42	1.41	0.49	−0.20	2.45	0.06	1.43
**SST / GNGT**	1.17	0.73	−1.69	3.23	<0.01	3.46	−0.24	0.81	−1.66

meanRR = mean interbeat interval duration; RMSSD = root mean square of successive difference; LF = low frequency power in HRV power spectrum; HF = high frequencies power in HRV power spectrum; LF/HF = ratio of low frequencies on high frequencies; E_i = entropy index; MFI = multifractal index. SCWT = Stroop color and word task; GNGT = go/no-go task; SST = stop signal task. * Indicates that distribution of feature values violated at least one parametric test assumption. In that case, nonparametric tests were conducted.

Table 2. - Interpretation scale of log (BF_{10}) factor based on Jeffreys 1961.

	Log (BF_{10})	Interpretation	Symbol
Growing evidence in favor of H_1	> 2	extreme evidence for H_1	H_1^{****}
	[1.48 ; 2]	very strong evidence for H_1	H_1^{***}
	[1 ; 1.48]	strong evidence for H_1	H_1^{**}
	[0.48 ; 1]	moderate evidence for H_1	H_1^{*}
	[0 ; 0.48]	anecdotal evidence for H_1	ns
	0	no evidence	ns
Growing evidence in favor of H_0	[−0.48 ; 0]	anecdotal evidence for H_0	ns
	[−1 ; −0.48]	moderate evidence for H_0	H_0^{*}
	[−1.48 ; −1]	strong evidence for H_0	H_0^{**}
	[−2 ; −1.48]	very strong evidence for H_0	H_0^{***}
	< −2	extreme evidence for H_0	H_0^{****}

BF_{10} = bayes factor; ns = non-significant; H_0 = null hypothesis; H_1 = alternative hypothesis. Interpretation scale: ****, ***, ** and * mean respectively, extreme, very strong, strong and moderate evidence for the null or the alternative one.

Supplementary reference:

Jeffreys, H. *Theory of Probability*, 3rd ed.; Oxford University Press: Oxford, United Kingdom, 1961.
